# Neurogenic Stunned Myocardium as a Complication of Aneurysmal Subarachnoid Hemorrhage: A Case Report and Lessons for Clinical Practice

**DOI:** 10.7759/cureus.90074

**Published:** 2025-08-14

**Authors:** Ana S Legarreta-Delgado, Javier Elizondo-Ramirez, Jesús A Jacquez-Graff, Jose C Herrera-Castro, José C Hernández-Pedroza, Luis A Ordoñez-Solorio, Arturo Muñoz-Cobos

**Affiliations:** 1 Medical School, Universidad Autónoma de Chihuahua, Chihuahua, MEX; 2 Neurosurgery, NeuroteamCUU (Chihuahua) Hospital Ángeles Chihuahua, Chihuahua, MEX; 3 Neurosurgery, Instituto Nacional de Neurología y Neurocirugía "Manuel Velasco Suárez", Mexico City, MEX; 4 Critical Care, NeuroteamCUU (Chihuahua) Hospital Ángeles Chihuahua, Chihuahua, MEX; 5 Neurological Surgery, NeuroteamCUU (Chihuahua) Hospital Ángeles Chihuahua, Chihuahua, MEX; 6 Neurological Surgery, NeuroteamCUU (Chihuahua) Hospital Star Médica Chihuahua, Chihuahua, MEX

**Keywords:** aneurysm, basilar artery, basilar artery bifurcation, neurogenic stunned myocardium, subarachnoid hemorrhage

## Abstract

Neurogenic stunned myocardium (NSM) is an entity resulting from two factors: left ventricular dysfunction coupled with a neurological condition. In our case, the state of our patient complicated things; she was fatally suffering from a Hunt and Hess grade 2 subarachnoid hemorrhage (SAH), which is not a common scenario. A 62-year-old female patient presented to the emergency department with an intense headache, dysarthria, and left hemiparesis that was attributed to CT findings of a Fisher grade 2 SAH, and the computed tomography angiography (CTA) also showed evidence of a ruptured aneurysm at the basilar artery bifurcation and an incidental aneurysm in the lower third of the basilar artery. NSM and SAH are not a common combination that must be considered for the unpredictable pathway of SAH management. This case underscores the need for early cardiac monitoring in SAH patients, as timely recognition and management of NSM may influence outcomes.

## Introduction

Neurogenic stunned myocardium (NSM) is a challenging entity that occurs only in approximately 30% of selected patients with subarachnoid hemorrhage (SAH). Its pathophysiological basis involves a sudden surge of catecholamines through activation of the hypothalamic-pituitary-adrenal axis and the sympathetic nervous system, leading to direct myocardial injury, microvascular dysfunction, and transient contractility impairment. These cases must be studied thoroughly with troponin measurement and echocardiography, as some may exhibit takotsubo syndrome (TTS), characterized by apical ballooning, occurring in only 1% to 6% of cases, which is associated with a worse outcome [1]. Typical NSM features include segmental wall motion abnormalities on echocardiography that do not follow a single coronary artery territory, reduced ejection fraction, and dynamic electrocardiographic changes.

The diagnosis of NSM is supported by the presence of an acute neurological event, new-onset left ventricular systolic dysfunction confirmed by echocardiography, elevation of cardiac biomarkers such as troponin and B-type natriuretic peptide (BNP or NT-proBNP), and exclusion of obstructive coronary artery disease as the primary cause. 

We discuss the case of a 62-year-old female patient who presented to the emergency department with clinical symptoms correlating to CT and computed tomography angiography (CTA) findings of a Hunt & Hess grade 2 SAH due to a ruptured aneurysm localized at the basilar artery bifurcation. The aim of this case report is to describe an uncommon presentation of NSM following SAH from a ruptured basilar artery bifurcation aneurysm, emphasizing the importance of early cardiac monitoring and multidisciplinary management.

## Case presentation

A 62-year-old female patient presented to the emergency department with a one-day history of multiple episodes of vomiting and a severe headache. Her past medical history included arterial hypertension diagnosed six months prior, managed with enalapril, and hospitalization for meningitis 32 years ago without sequelae. She reported smoking seven cigarettes per day since the age of 13.

On arrival, her vital signs revealed a blood pressure of 189/110 mmHg, a heart rate of 88 beats per minute (bpm), a respiratory rate of 18 breaths/minute, a temperature of 36.7°C, and an oxygen saturation of 96% on room air. Neurological examination showed dysarthria and left hemiparesis with a Daniel’s score of 4/5, corresponding to Hunt & Hess grade 2. The remainder of the systemic examination was unremarkable. Cardiovascular examination revealed regular heart sounds without murmurs, gallops, or rubs, and no signs of heart failure.

**Figure 1:**
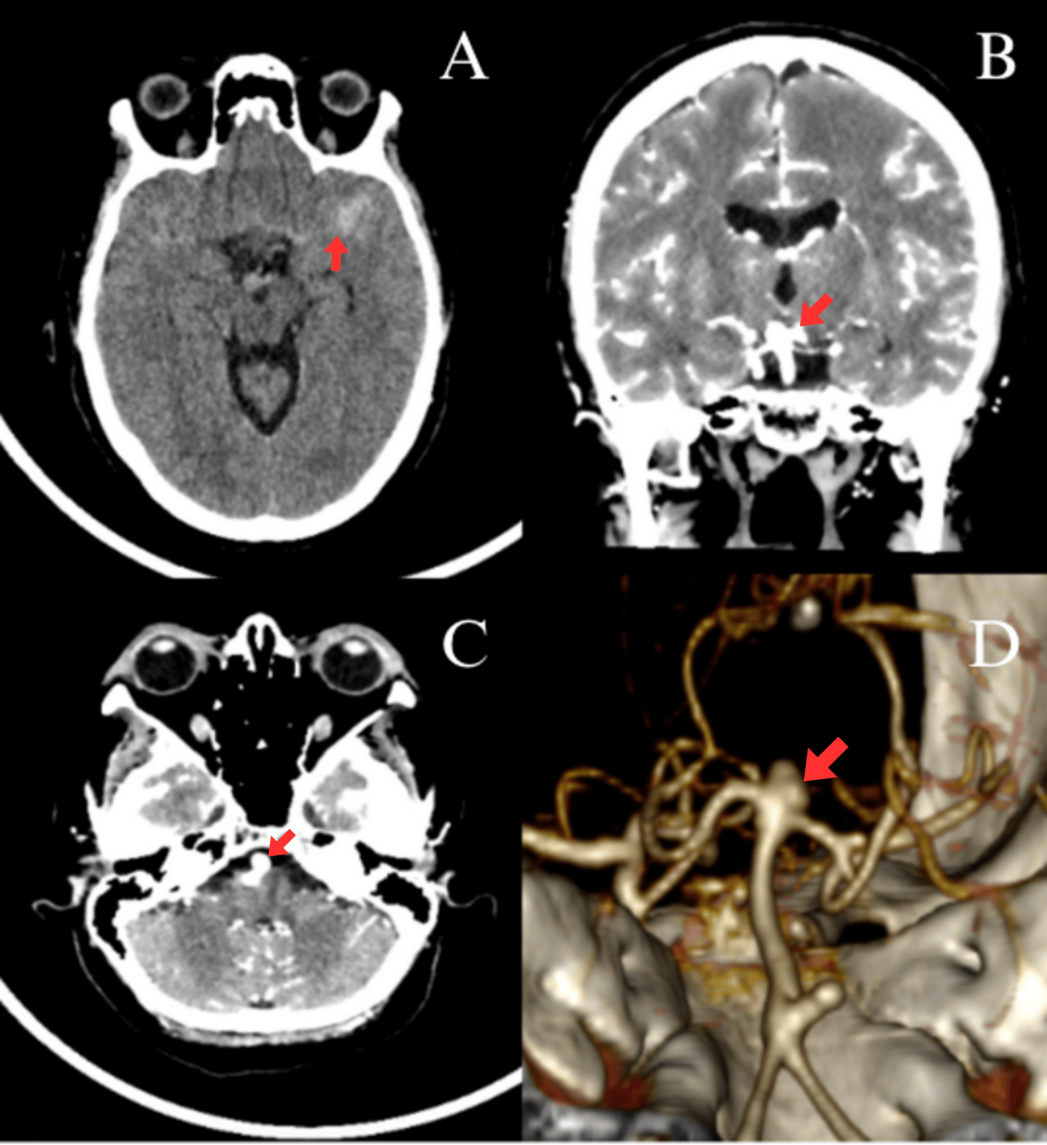
A) Fisher grade 2 subarachnoid hemorrhage; B) Coronal slice showing aneurysm at the basilar artery bifurcation; C) Axial slice displaying aneurysm in the lower third of the basilar artery; D) 3D reconstruction showing both aneurysms of the basilar artery; the red arrow shows the treated aneurysm in this case.

A non-contrast cranial CT scan demonstrated a Fisher grade 2 SAH. CTA identified a ruptured aneurysm at the basilar artery bifurcation and an incidental aneurysm in the lower third of the basilar artery. Given the findings and the lack of resources at the initial facility, she was transferred to our hospital for definitive management (Figure 1).

**Figure 2:**
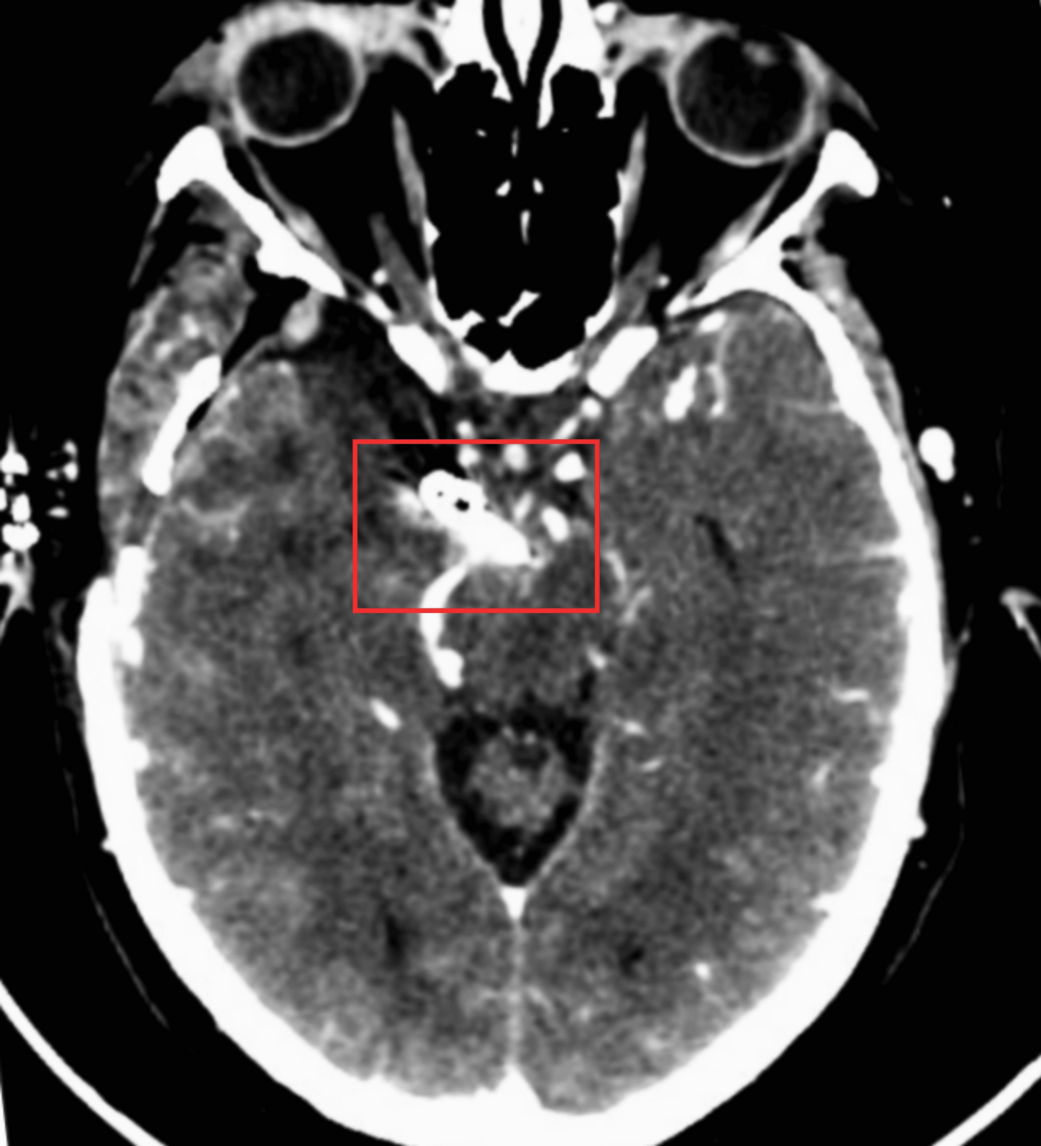
Postoperative control cranial CT scan showing an aneurysm clipping at the basilar artery bifurcation (red square).

Three days after symptom onset, the patient underwent a pretemporal craniotomy with microsurgical clipping of the ruptured aneurysm, without intraoperative complications. She was admitted to the intensive care unit (ICU) for postoperative monitoring (Figure 2).

During her ICU stay, transcranial Doppler evaluation of both middle cerebral arteries revealed a mean flow velocity >160 mm/s and a Lindegaard index >6, consistent with severe vasospasm. The patient received intravenous nimodipine (2 mg/h), norepinephrine infusion titrated to maintain a mean arterial pressure >100 mmHg, and prophylactic levetiracetam (500 mg every 12 hours). Despite these measures, the vasospasm persisted, leading to intra-arterial nimodipine administration via pharmacological angioplasty in the catheterization laboratory.

Following critical care protocols, a transthoracic echocardiogram was performed, showing an ejection fraction of 20% (biplane method), inferior and medial wall hypokinesis with basal segment hyperkinesis, left atrial dilation, grade 3 diastolic dysfunction, and no valvular abnormalities. The electrocardiogram revealed sinus rhythm without ischemic changes. Serial biomarkers showed a rise in troponin I from 0.02 ng/mL at admission to 0.96 ng/mL at 24 hours and 1.12 ng/mL at 48 hours, with BNP measured at 1,350 pg/mL on ICU day 2.

Given the echocardiographic abnormalities, elevated cardiac biomarkers, absence of a history of ischemic heart disease, and the clear temporal association with SAH, the diagnosis of NSM was established. Supportive therapy with levosimendan (0.1 μg/kg/min) was initiated on ICU day 4 to address cardiac failure. Despite this, the patient’s condition deteriorated, with progressive hemodynamic instability requiring dual vasopressor support. Placement of an intra-aortic balloon pump was contemplated; however, before the intervention could be undertaken, the patient developed brain death secondary to severe and refractory cerebral vasospasm.

## Discussion

Reversible left ventricular dysfunction known as stunned myocardium, coupled with neurological pathologies, leads to NSM, characterized by electrocardiographic and echocardiographic abnormal changes, troponin, as well as wall motion disturbances, such as hypokinesis, the segmented contractility alteration may affect any left ventricular area, particularly, on the anterior, septal and apical level, causing a systolic dysfunction with oscillating ejection fractions between 10 and 50%, also, with less frequency, right ventricular dysfunction may appear. All of these, as mentioned earlier, have been reported in patients with NSM after SAH [2-4].

On ICU admission, the patient’s Sequential Organ Failure Assessment (SOFA) score was nine, and the Acute Physiology and Chronic Health Evaluation II (APACHE II) score was 21, indicating high predicted mortality. The Society for Cardiovascular Angiography and Interventions (SCAI) Cardiogenic Shock Classification [5] placed the patient at stage C (classic), consistent with the presence of hypotension requiring vasopressor support and evidence of end-organ hypoperfusion.

As proposed by Goico et al., severe stress may activate the hypothalamic-pituitary-adrenal axis, leading to elevated catecholamine levels. As a result, their effect on cardiomyocytes produces a “stunned myocardium” through β1-adrenergic receptors. Norepinephrine has a positive inotropic response, primarily affecting the inferior left wall of the heart. Additionally, β2-adrenergic receptors, when stimulated by epinephrine, usually have a positive effect on heart function at normal levels. However, if epinephrine levels are too high, it can cause the heart to weaken instead, essentially impacting the left ventricular apex [6].

Catecholamines promote vasospasm, increasing cardiac workload. This leads to a supply-demand mismatch and post-ischemic myocardial stunning [7,8]. As in our case and the revision we have made, we can associate the vasospasm generated by systemic stress originating from the SAH. According to Kerro et al., a Hunt & Hess scale greater than two predicts a worse outcome. Nevertheless, this case presents as a Hunt & Hess grade 2, which does not associate with the severity [2]. Other risk factors present include an aneurysm in the posterior circulation and female gender. We found five cases in which stunned myocardium and brain aneurysms coexisted, and it is interesting how three out of five were located at the tip of the basilar artery. A total of four female patients (80%) were retrieved, in which biomarkers coincided with an elevated parameter. All patients had a low ejection fraction except for case number one, which was borderline (45%). Echocardiography revealed nearly identical findings with slight variations in wall segments. The ECG shows variable but expected changes, including “camel hump” T waves, T wave inversions, QT prolongation, and ST elevation. Surgical and endovascular management procedures included aneurysm clipping and aneurysm embolization by coiling. ICU management consisted of heart failure pharmacological control, and in some cases, vasospasm pharmacological management was also employed.

Echocardiography revealed a markedly reduced ejection fraction with regional wall motion abnormalities, and the ECG did not show significant changes. Serial biomarkers revealed a progressive increase in cardiac enzymes: troponin I rose from 0.02 ng/mL on admission to 0.96 ng/mL at 24 hours and 1.12 ng/mL at 48 hours. BNP was measured at 1,350 pg/mL on ICU day 2. These findings support the diagnosis of acute cardiac dysfunction consistent with NSM. Elevated levels of creatine phosphokinase (CPK) or creatine kinase-MB (CK-MB) isoenzyme, significant troponin release, and increased levels of BNP suggest cardiotoxic myocardial dysfunction, which can lead to myocardial necrosis [3,4]. 

Differential diagnosis can be made with myocarditis, which presents with clinical conditions similar to myocardial stunning; acute coronary syndrome; parallel electrocardiographic abnormalities, such as ST-segment depression or elevation, T-wave inversion and Q waves; echocardiographic changes, enzymatic rise, and CPK-MB elevations, nonetheless, troponin rise is higher in this pathology, TTS; typical TTS involves an emotional and/or physical stress factor, and it also prevails in women, with similar ECG disturbances, as well as prolonged QT-interval, ST-segment depression (new onset), ST-segment elevation, various arrhythmias, paroxysmal or persistent atrial fibrillation, and U-waves [3,4,9]. 

As indicated by the Hunt-Hess grade, the severity of NSM may be associated with the severity of SAH; this neurologic injury degree acts as a risk factor after SAH, accompanied by female gender. Delving into acute myocardial dysfunction, which is often accompanied by brain injury, reveals increased plasma levels of epinephrine and norepinephrine, as well as alterations in β-adrenergic signal transduction [2,3].

Typically, an abnormal ECG pattern appears, consisting of large inverted T waves, prolonged QT intervals, and large U waves. If hyperkalemia is detected, tall T waves may appear; conversely, U waves are typically associated with hypokalemia [3,10].

The pathogenesis of NSM involves a complex interconnection of neurological, endocrine, and cardiovascular mechanisms. This condition primarily arises from significant stressors, such as neurological injuries, that initiate a cascade of events involving the central nervous system and the adrenergic autonomic nervous system. These stressors, particularly those that damage the insular cortex and parietal lobe, disrupt cardiovascular regulation and lead to the excessive release of catecholamines from multiple sources, including the adrenal glands, myocardium, and the locus coeruleus [2,3].

The overstimulation of β-adrenergic receptors by these catecholamines triggers several pathological effects in the cardiovascular system. These include increased myocardial contractility and a mismatch between oxygen supply and demand, resulting in microcellular hypoxia, mitochondrial dysfunction, and eventual myocardial injury or cell death [2,3].

Furthermore, the diencephalon and brainstem nuclei play critical roles in autonomic cardiovascular regulation. This sympathetic hyperstimulation not only leads to cardiac dysfunction but also contributes to coronary vasospasm and ischemia [2,3,11].

In addition to the sympathetic effect, the parasympathetic nervous system modulates the myocardial inflammatory response via acetylcholine receptors, creating a complex dynamic between injury and recovery. The prolonged elevation of circulating catecholamines (up to 10 days after events such as SAH) highlights the persistent impact of this hyperadrenergic state, highlighting its role in the pathogenesis of myocardial damage observed in NSM [2,3].

In this case, intensive care management focused on hemodynamic stabilization, vasospasm control, and support for acute heart failure, consistent with current recommendations for SAH complicated by NSM. Intravenous nimodipine was administered to prevent and treat cerebral vasospasm, while norepinephrine was titrated to maintain adequate cerebral perfusion pressure (mean arterial pressure >100 mmHg). Prophylactic levetiracetam was used to reduce the risk of seizure-related secondary injury. Levosimendan, a calcium sensitizer with inotropic and vasodilatory properties, was initiated on ICU day 4 to address severe systolic dysfunction refractory to standard measures. Despite these interventions, the patient’s condition progressed to brain death due to severe, refractory vasospasm, highlighting the potential limitations of optimal medical therapy in advanced cases of SAH-associated NSM.

For patients with SAH, treatments should include prevention of rebleeding with early aneurysm clipping or endovascular treatment focused on the underlying neurologic condition [3]. A condensed summary of similar cases appears in Table 1. 

**Table 1:** Stunned myocardium cases related to aneurysmal SAH SAH: subarachnoid hemorrhage; CTA: computed tomography angiography; LVSF: left ventricular systolic function; ACEI: angiotensin-converting enzyme inhibitors; EF: ejection fraction; DSA: digital subtraction angiography; IABP: intra-aortic balloon pump

Variables	Case 1	Case 2	Case 3	Case 4	Case 5
Author	Goico et al. [5]	Kerro A et al. [2]	Franco C et al. [10]	Lampropoulos K et al. [1]	Ono Y et al. [11]
Age (years)	55	75	Adult	41	66
Sex	Female	Female	Female	Female	Female
Biomarkers (ng/mL)	Initial normal, then 20x	Initial <0.02, then 72x	4.2	Elevated	Positive
Ejection fraction (%)	45	Mild to moderate reduction	20	25	28
Hypokinetic wall segments	Inferior, inferolateral, mid-lateral, inferior septal	Apical	Apical, distal left	Apical and mid-ventricular	Severe hypokinesia (apical, inferior, and anterior) of the left ventricle
ECG findings	Initial ‘‘camel-hump’’ T-waves, then T-wave inversions		T wave inversion with prolonged QT	No changes	T wave inversion and ST elevation
Aneurysm (mm)	Not mentioned	6.5	3	2	Not specified
Aneurysm location	Basilar artery tip	Basilar artery tip	Basilar artery tip	Supraclinoid internal carotid artery	Middle cerebral artery bifurcation
Diagnosis	CT (SAH), CTA (ruptured aneurysm), echocardiogram (45% EF)	CT (SAH), CTA (ruptured aneurysm), echocardiogram (mild to moderate reduction in LVSF)	CT (SAH), angiogram (ruptured basilar aneurysm), echocardiogram	CT (SAH), angiogram (no aneurysm), DSA	CT (SAH), echocardiogram
Treatment	Embolization, beta-blockers, inotropic agents, vasopressors, and an IABP	Coil embolization, catecholamines	Embolization, ACEI, beta-blockers, diuretics, and vasopressors	Digoxin, ivabradine, and diuretics, craniotomy, and clipping of an aneurysm	Catecholamines (rest not specified)
Outcome	Neurologic status improved, troponins and ECG normalized, hypokinesia and ventricular dysfunction resolved	Neurologic status improved gradually, prior left ventricular dysfunction and apical hypokinesis resolved	EF of 45%, no identifiable wall motion abnormality	“Very good recovery”	Two months later was discharged with normalized heart function

## Conclusions

NSM can occur even in moderate cases (Hunt & Hess grade 2), specifically in posterior circulation aneurysms. Catecholamine storms can cause fatal cardiac dysfunction. Early cardiac monitoring and imaging are important independently of every case scenario of subarachnoid hemorrhage to identify NSM and prevent hemodynamic collapse. A multidisciplinary team is necessary to effectively manage patients of this type. 

This case highlights the importance of systematic cardiac assessment in patients with SAH. Early recognition of NSM, guided by biomarkers and echocardiography, may guide timely interventions and improve outcomes. Multidisciplinary collaboration between neurosurgery, cardiology, and critical care is essential in managing such complex cases. Further research is needed to determine whether the location of an aneurysm affects the outcome after its rupture.
